# An unusal case of coarctation associated with hypoplasia of the aortic arch and tissue paper aortic wall thickness: a difficult surgical problem

**DOI:** 10.1186/1749-8090-8-S1-P98

**Published:** 2013-09-11

**Authors:** H Rodriguez, Y Heredia, E Brenner, V Yakutis, J Ramirez, I Morel, T DiSessa

**Affiliations:** 1 International Children’s Heart Foundation, Monterrey, Mexico; 2 Hospital Arturo Grullon, Santiago, Dominican Republic; 3 International Children’s Heart Foundation, Cincinnati, OH, USA; 4 International Children’s Heart Foundation, Belarus; 5 International Children’s Heart Foundation, Lexington, KY, USA

## Background

Coarctation of the aorta is frequently associated with aortic arch hypoplasia. This combination is more frequent in neonates, often with severe symptoms and in critical condition. We report an isolated case of aortic arch hypoplasia with tissue paper aortic wall and subclavian artery aneurysm in a young adult that was repaired successfully.

## Methods

Single isolated case; we reviewed the record and pre/ post-surgical CT scan.

## Results

Patient is a male age 21 yo, without relevant past medical history and class I NYHA. Six months prior to diagnosis presented he several episodes of syncope like symptoms and chest pain, that limited his physical activity. Preemployment medical evaluation revealed a systolic murmur III / VI throughout the precordium with radiation to the neck, pressure in upper extremities of 190/100 mmHg and in lower limbs of 90/60 mmHg and cardiomegaly. CT scan with IV contrast showed hypoplasia of the aortic arch, left subclavian artery aneurysm and coarctation of the aorta. The echocardiogram also revealed a ventricular septal defect and a subaortic diaphragm. Corrective surgery was performed by median sternotomy, aortic and femoral cannulation and deep hypothermia with antegrade cerebral perfusion. Ventricular septal defect was closed with PTFE patch, subaortic membrane was resected and the reconstruction of the aortic arch and isthmus was done with a Goretex graft number 20. We left the subclavian artery isolated (without reimplantation) since there was sufficient retrograde flow. Pre and Postoperative angio CT scan are shown. The patient returned to his normal life and his blood pressure is normal 6 months after surgery.

## Conclusion

Corrective surgery of the unusual adult presentation of aortic arch hypoplasia and coartaction of aorta is feasible and provides good results and effective relief of symptoms.

